# Opioidergic Modulation of Striatal Circuits, Implications in Parkinson's Disease and Levodopa Induced Dyskinesia

**DOI:** 10.3389/fneur.2018.00524

**Published:** 2018-07-05

**Authors:** Stefania Sgroi, Raffaella Tonini

**Affiliations:** Neuromodulation of Cortical and Subcortical Circuits Laboratory, Neuroscience and Brain Technologies Department, Istituto Italiano di Tecnologia, Genoa, Italy

**Keywords:** opioids, dopamine, striatum, Parkinson's disease, signaling pathway, synaptic plasticity

## Abstract

The functional organization of the dorsal striatum is complex, due to the diversity of neural inputs that converge in this structure and its subdivision into direct and indirect output pathways, striosomes and matrix compartments. Among the neurotransmitters that regulate the activity of striatal projection neurons (SPNs), opioid neuropeptides (enkephalin and dynorphin) play a neuromodulatory role in synaptic transmission and plasticity and affect striatal-based behaviors in both normal brain function and pathological states, including Parkinson's disease (PD). We review recent findings on the cell-type-specific effects of opioidergic neurotransmission in the dorsal striatum, focusing on the maladaptive synaptic neuroadaptations that occur in PD and levodopa-induced dyskinesia. Understanding the plethora of molecular and synaptic mechanisms underpinning the opioid-mediated modulation of striatal circuits is critical for the development of pharmacological treatments that can alleviate motor dysfunctions and hyperkinetic responses to dopaminergic stimulant drugs.

## Introduction

Opioidergic signaling is involved in several functional aspects of the peripheral and central nervous system and due to the broad distribution of opioid receptors throughout the brain, its activation modulates different neural circuits. Opiate drugs are widely used as analgesic to induce antinociception and to treat pain disorders. However, edonic effects of opiates induce addictive behaviors that entail the involvement of opioidergic system in reward processes (1, 2). Opioid receptors and the endogenous opioid peptides Enkephalin (Enk) and Dynorphin (Dyn) are expressed at striatal circuits, where the opioid system modulates the activity of spiny projection neurons (SPNs) during movement control in both a healthy state and in motor disorders such as Parkinson's disease (PD). In PD, functional changes in striatal pathways are associated with a reorganization of molecular and synaptic mechanisms that counteract the loss of dopaminergic cells. However, aberrant neuroadaptations in the striatal circuit can be responsible for critical aspects of PD, as observed in levodopa-induced dyskinesia (LID). It is still unclear what role opioid transmission plays in striatal circuity and how this system affects neural reorganization, both in PD and in response to dopaminergic treatment. Here, we review recent findings on the cell-type-specific effects of opioid transmission in the dorsal striatum, including the signaling pathways, synaptic and behavioral effects mediated by opioid ligands, as well as their interactions with dopaminergic transmission in both a PD state and in response to dopaminergic treatment with levodopa (L-DOPA).

## Anatomy and physiology of the basal ganglia

The basal ganglia (BG) comprise a distributed group of nuclei that include the striatum, which is composed by the caudate and putamen (CPu), the globus pallidus, with the pars externa (GPe) and interna (GPi), the subthalamic nucleus (STN); and the substantia nigra pars compacta (SNpc) and pars reticulata (SNpr). The Striatum and the STN represent the main input nuclei of the BG, while the GP and SNpr are the two output structures projecting to the thalamus and brainstem (3–7). The BG nuclei's connectivity to cortical regions provides a complex network of sensorimotor, limbic and associative information, conferring on the BG a pivotal role in the control of movement as well as in associative learning, emotion and reward-related behavior (8).

**Figure 1:**
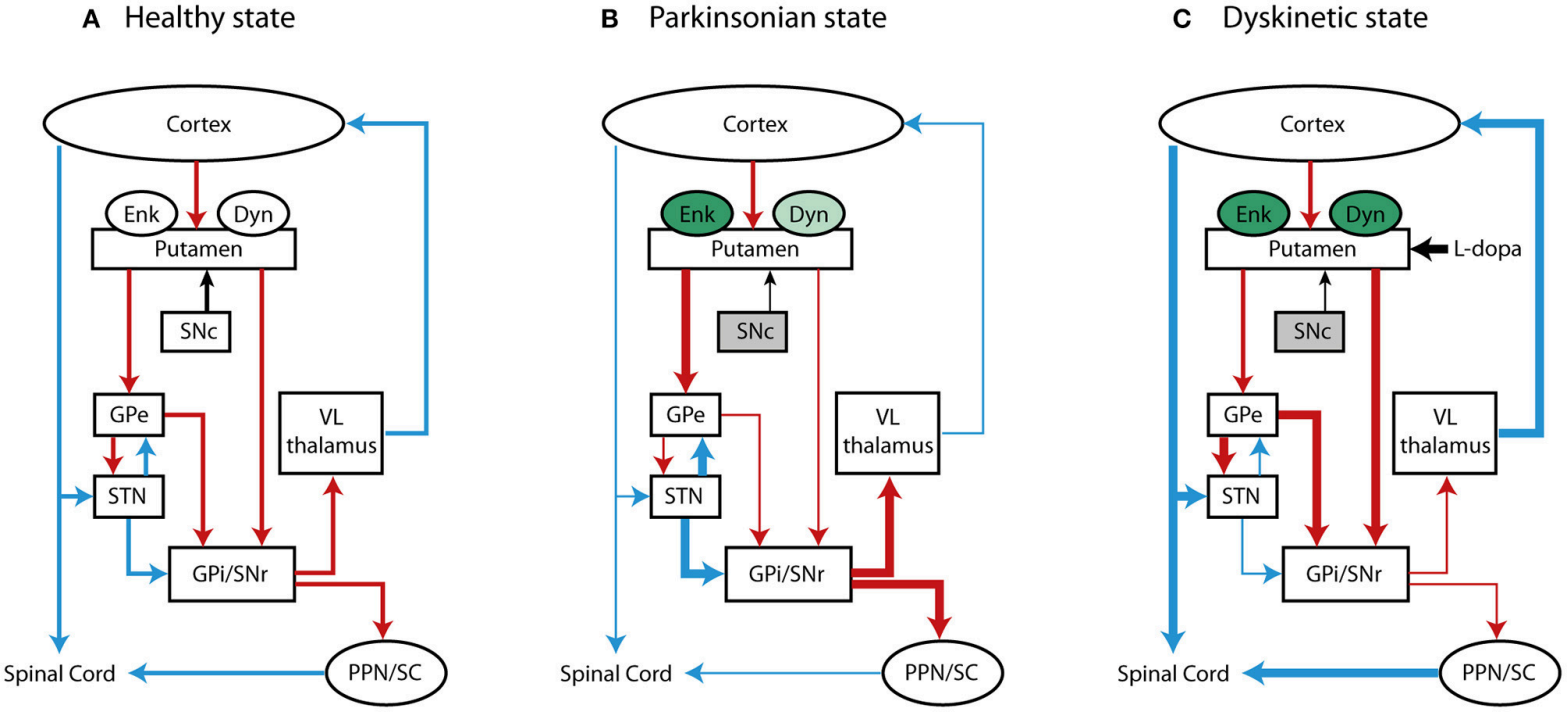
Basal ganglia motor loop in **(A)** normal, **(B)** PD, and **(C)** LID condition. The schematic represents the direct, indirect and hyperdirect pathways projecting to the thalamus and spinal cord and the changes of expression of opiod peptides, Enk and Dyn. Red, blue and black lines indicate GABA-ergic, glutamatergic and dopaminergic projections, respectively. Changes in the rate of neural transmission are indicated with thick (increased activity) and thin (decreased activity) lines. Changes in the expression of Enk and Dyn are depicted in green (increased levels) and light green (decreased levels). The gray color of substantia nigra pars compact (SNc) is representative of PD state due to the loss of dopaminergic cells.

Nearly 95% of the striatum is composed of striatal projection neurons (SPNs), which are GABA (γ-aminobutyric acid)-ergic neurons that relay inhibitory efferent transmission and are rich in dopaminergic receptors (DR). These neurons are classified in two subtypes based on their projection targets, neuropeptides expression and DR subtypes (9). SPNs that express the neuropeptide Dyn and bear D1 excitatory receptors (D1Rs) (10) belong to what is termed the direct striatonigral pathway (dSPNs), projecting directly to the GPi/SNpr. On the other hand, SPNs expressing Enk and bearing D2 inhibitory receptors (D2Rs) project to the GPi/SNpr indirectly through the GPe, as part of the indirect striatopallidal pathway (iSPNs) (9, 11). In a healthy state (see Figure 1A), the activation of the direct pathway promotes movement execution by reducing the neural firing of the GPi/SNpr to the thalamus and boosting glutamatergic thalamocortical transmission. In parallel, activation of the indirect pathway reduces movement initiation, exciting GPi/SNpr transmission by inhibiting the GPe and activating the STN, ultimately leading to the inhibition of thalamocortical transmission (4, 12, 13). The concomitant activation of both striatofugal pathways maintains a balance between the direct and indirect pathways, activating specific and voluntary actions through the direct pathway and inhibiting involuntary movements through the indirect pathway (13, 14).

Excitatory corticostriatal transmission on SPNs is modulated by dopaminergic input from the SNpc through “diffusion-based volume transmission,” where dopamine (DA) diffuses away from the synapse to reach extrasynaptic receptors and regulate excitability of SPNs (15). However, sparse release sites defined as active zone have been identified in the striatal DA axons to allow for a fast DA release and to generate a localized DA signal (16). Once released, DA exerts a dual effect on striatal neurons (17), exciting the direct pathway by binding to D1Rs and inhibiting the indirect pathway by binding to D2Rs. DA discharge from the dopaminergic neurons of the SNpc is crucial for the initiation and execution of motor sequences (14, 18).

## The opioidergic system: peptides and receptors

Enk, Dyn and β-endorphin belong to family of endogenous peptides produced through the proteolytic cleavage of protein precursors such as preproenkephalin-A (PPENK), which forms six copies of methionine-Enk (Met-Enk) and one copy of leucine-Enk; preproenkephalin-B (also known as preprodynorphin), which produces Dyn and endorphin; and finally, proopiomelanocortin, which produces β-endorphin. The endogenous peptides have different degrees of selectivity for the opioid receptors; Enk binds δ opioid receptors (DORs) and μ opioid receptors (MORs), Dyn is selective for κ-opioid receptors (KORs), and β-endorphin binds MORs (1).

Opioid receptors (ORs) are seven-transmembrane receptors and belong to a superfamily of G protein-coupled receptors (GPCRs) with inhibitory activity (Gα_i/o_) on cellular excitability and synaptic transmission (1). OR activity promotes the activation of G-protein-coupled inwardly rectifying K^+^ channels, inhibits Ca^+^ channels and adenylyl cyclase (AC), and reduces neurotransmitter release and neural activity (19, 20). ORs are broadly distributed in the brain, with some structures exhibiting higher expression of a specific type of receptor, while others have three overlapping receptors that can interact locally with one another in synergistic or antagonistic ways (21).

**Figure 2:**
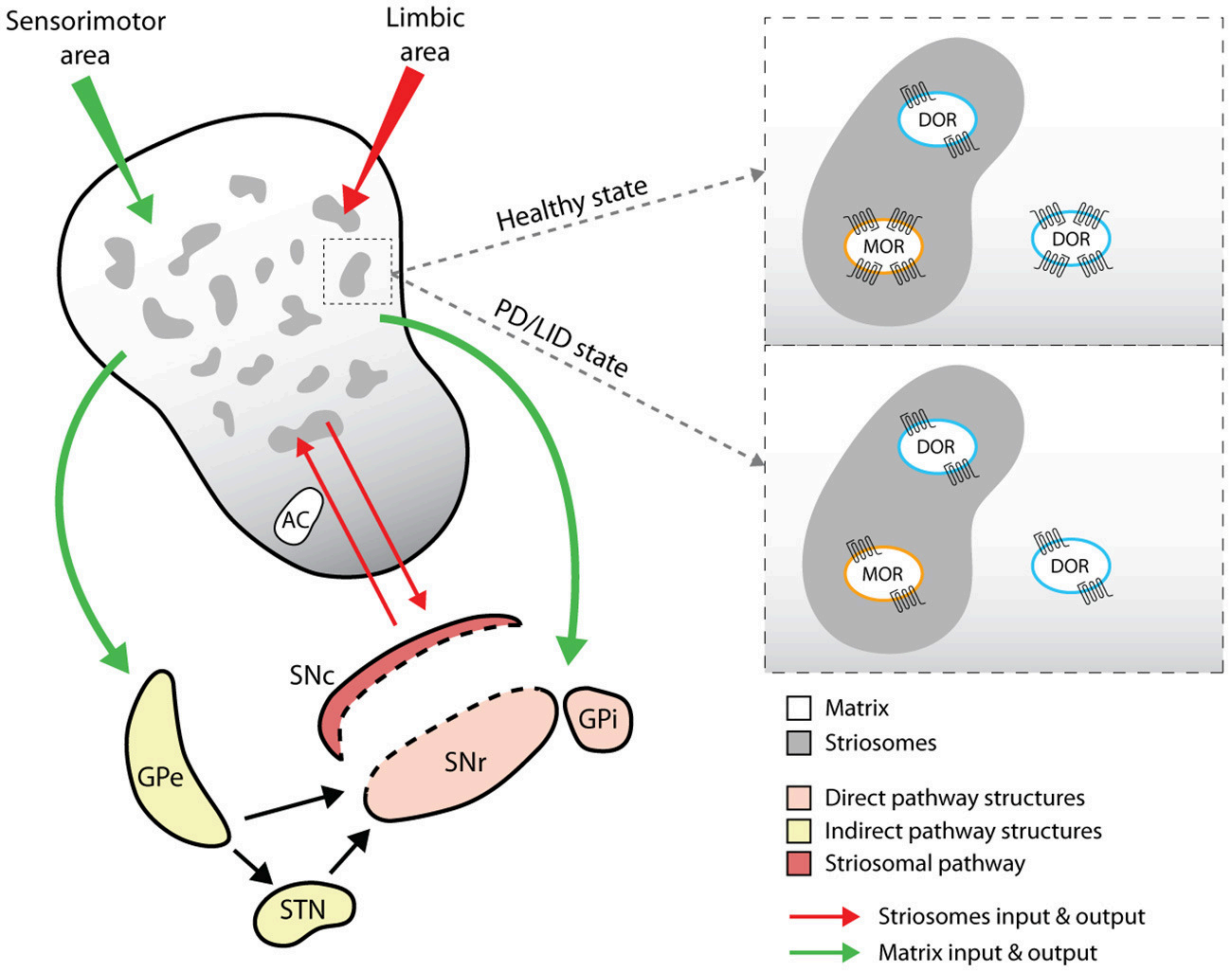
Representative cartoon of opioid receptors distribution and expression in striosomes and matrix compartments of the dorsal striatum. The drawing shows the different input and output pathways related to the striosomes (red lines) and matrix compartments (green lines) and the changes in the expression levels of MOR and DOR. GPe (globus pallidus pars externa) and STN (subthalamic nucleus) indicate the indirect pathway targets; SNr (substantia nigra pars reticulata) and GPi (globus pallidus pars interna) indicate the direct pathway targets; SNc (substantia nigra pars compacta) is the target of the specific pathway arising from the striosomal compartments.

A broad distribution of MORs has been observed in the thalamus, amygdala and locus coeruleus (1), and in the thalamic afferents to the striatum (22). MOR expression has also been observed in CPu striosomal compartments that project to the SNpc (23) (Figure 2). Specifically, MORs are expressed in striosomes both on dSPNs enriched in Dyn precursor and on iSPNs (24), where they colocalize with D2Rs in dendrites (25). MORs are also expressed on striatal cholinergic interneurons (26, 27).

DORs are abundant in layers II, III, IV and V of the cerebral cortex and in the striosomes and matrix compartments of the CPu, with a higher dorsolateral distribution than ventromedial (28). On a cellular level, DORs are expressed within the nucleus and in the somatodendritic area on iSPNs, but not on dSPNs (24, 29).

KORs are predominantly found in the medial sector of the CPu and in the nucleus accumbens and showed a higher coexpression with D1Rs (24). They are also localized presynaptically in the nigrostriatal afferents of the SNpc.

## Opioid receptor signaling

The activation of opioidergic GPCRs by endogenous opioid peptides or exogenous agonists leads to the dissociation of Gα/βγ subunits that stimulate various intracellular effectors. The inhibitory activity of opioid receptors includes several processes that are selectively initiated by the Gα and Gβγ protein subunits. The **G**α subunit inhibits AC by decreasing intracellular cyclic adenosine monophosphate (cAMP) levels and activates the inward-rectifier K^+^ channel, leading to the hyperpolarization of the cellular membrane and the inhibition of neural activity (30). The inhibition of AC and cAMP by the Gα subunit can also lead to a reduction of Ca^2+^ conductance (31), although this reduction is predominately induced by the direct binding of **G**βγ subunit to the channel, and the consequent decrease in neurotransmitter release. Indeed, activation of KORs on the nigrostriatal afferents of the SNpc reduces the release of DA and influences the kinetics of the DA transporter (32, 33). Intrastriatal injection of a MOR agonist alters extracellular DA levels in the shell and core of the nucleus accumbens and in the rostral and caudal subregions of the dorsal striatum, where the reduction is thought to be due to presynaptic activation of MORs on DA terminals (34, 35). Activation of MORs on striatal cholinergic interneurons reduces Ach release and decreases cholinergic interneurons excitability (26, 27).

Besides inhibiting the AC /cAMP, opioid receptors shape several other cellular responses. The interaction with different G proteins, β-arrestins or kinases, can promote the activation of different effectors or signaling pathways (36), or prompt the internalization and desensitization of receptor functional activity (19, 37), with significant changes in behavior (38). The direct activation of opioid receptors and the release of Gβγ subunits can promote the activation of mitogen-activated protein kinases (MAPKs) (19, 39). Notably, activation of MAPK can be also mediated by binding of DA to D1R. In the dorsal striatum of PD animal models, pulsatile replacement of DA, for example by L-DOPA treatment, leads to an overstimulation of the direct striatonigral pathway that promotes the activation of MAPK and its downstream effectors, such as extracellular signal-regulated kinases ERK1/2 or transcription factors (40–42). Increased levels of phosphorylated ERK (p-ERK) or immediate early genes are associated with aberrant cellular responses and dysfunctional behaviors in PD and LID state (43–45). Therefore, opioidergic and dopaminergic receptors could both activate postsynaptic signaling cascades that converge to ultimately promote an increase of proteins and transcriptional factors that affect striatal-based behaviors. However, it is still unclear whether alterations of the striatal motor function arise from a synergic activity of the dopaminergic and opioidergic system or if opioid transmission only modulates the molecular and synaptic mechanisms mediated by dopaminergic transmission.

## Compartment-specificity localization of opioid receptors in the dorsal striatum

Beyond the classical division of the striatum into the direct and indirect pathways, this structure is also subdivided into striosomes (defined as striatal bodies) and matrix compartments (Figure 2), which are defined according to neurotransmitter and receptor segregation, afferent and efferent connections (46), signaling cascade activation (47) and neurophysiological features (48). Striosomes represent about 10–15% of the dorsal striatum and are mainly localized in the medial sector of the CPu (29, 49), where they are characterized by acetylcholinesterase (AchE)-poor zones and by immunoreactivity against Enk, substance P and GABA (50). The matrix compartment comprises 85% of the remaining striatum. It is rich in AchE, contains calcium-binding proteins such as parvoalbumin and calbindin, and is directly affiliated with the sensorimotor system (51). Both striosomes and matrix contain dSPNs and iSPNs, although dSPNs are more prevalent in the striosomal compartment and project predominantly to dopaminergic neurons in the SNpc (50, 52).

The matrix and striosomal compartments also receive inputs from different cortical areas; striosomes are related to the limbic area, whereas the matrix is associated with sensorimotor and associative areas (53) (see Figure 2). Overall, this complex striatal subdivision, together with a discrete distribution of neuromodulators between matrix and striosomes compartments, reflects that SPNs functional activity might differ in compartment-specific manner and affect different striatal-based behaviors (54).

While dSPNs and iSPNs are broadly distributed in both striosomes and matrix, opioid-mediated synaptic transmission seems to segregate (46), perhaps due to the different distributions of opioid receptors on dSPNs and iSPNs in these compartments. For example, application of MOR and DOR agonists reduces GABAergic synaptic responses in both dSPNs and iSPNs predominantly in the striosomal compartment, but not in the matrix (29, 48). Specifically, the binding of Enk to DORs located on iSPNs collaterals that synapse on dSPNs, suppresses the inhibition of dSPNs only in the striosomes, but not in matrix, leading to strengthened striosomal dSPNs responses to corticostriatal inputs (29). The behavioral implications of this connectivity might be relevant in PD, where changes in the levels of the endogenous opioid Enk might promote or reduce dSPNs response to cortical inputs, thereby affecting the release of DA through the striatonigral pathway (29).

## Opioid-mediated neurotransmission and synaptic plasticity in the dorsal striatum

The first neurophysiological studies on the opioid-mediated neurostransmission at striatal circuits investigated the role of these neuropeptides in the modulation of glutamatergic inputs mainly arising from the cortex. These studies showed that MOR and DOR agonists inhibited glutamatergic inputs to the striatum (55) and more specifically, selective MOR agonists reduced the excitatory inputs at the corticostriatal level in both striosomes and matrix compartments (48, 56). In addition, the application of exogenous MOR and DOR agonists or the release of endogenous opioids induced long-term depression (LTD) on striatal SPNs in both the DLS and the dorsomedial striatum (DMS). Specifically, MOR activation inhibited thalamostriatal excitatory inputs, whereas the activation of DOR inhibited corticostriatal inputs; these results indicate the specificity of opioid-mediated synaptic plasticity in the dorsal striatum (22). Interestingly, applying an exogenous KOR agonist induced LTD more selectively in the DLS than in the DMS, suggesting subregional specificity of KOR-mediated synaptic plasticity (22) (Table 1). This subregional difference between the DLS and DMS might be related to their distinct functional roles in motor control. Behavioral studies demonstrate that the DLS is more connected to the control of body movements rather than to more general control of locomotor activity (58, 59). In PD animals treated with L-DOPA, higher levels of Dyn precursor (PDYN) mRNA, selective for KOR binding, are expressed in the DLS than the DMS (60–62). Moreover, higher PDYN mRNA expression in the lateral striatal portion of the DA-denervated hemisphere correlates only with the severity of dyskinesia, instead of with locomotor variables that define animals' spontaneous motion (60, 62).

**Table 1:** Summary of opioid-mediated neurotransmission and synaptic plasticity in the dorsal striatum.

**Activation of opioid recepetors**	**Distribution**	**Signal**
MOR	Thalamostriatal afferents Striosomal dSPN and iSPN Cholinergic interneurons	↓EPSCs (22) ↓IPSCs (29, 48) ↓ACh release (26, 27)
DOR	Nigrostriatal terminal iSPN striosomal collaterals Corticostriatal afferents	↓ DA release (34, 35) ↓ IPSCs (29) ↓ EPSCs (22)
KOR	Presynaptic nigrostriatal afferents Striatal SPNs in DLS	↓ DA release (33) LTP (57) LTD (22)

*According to the specific distribution of opioid receptors, changes in neurotransmitter release or synaptic plasticity are observed in response to exogenous and endogenous opioid agonists. ESPCs, excitatory postsynaptic currents; ISPCs, inhibitory postsynaptic currents; Ach, acetylcholine; DA, dopamine; LTP, long term potentiation; LTD, long term depression*.

Opioids have been shown to regulate striatal LTD (22). In contrast, their effect on long-term potentiation (LTP) in the dorsal striatum remains unexplored. Most of the studies that have attempted to characterize the role of opioids in LTP have examined different functional areas, such as the ventral tegmental area (63), hippocampus or C-fiber of the spinal dorsal horn (64). A recent study investigated the effect of KOR activation on LTP in the corticostriatal pathway (57), and demonstrated that applying Dyn reduced the release of DA, as expected by binding to KOR on DA nigrostriatal terminals. Moreover, selective activation of the D1R-SPNs that promote the co-release of Dyn also led to impared corticostriatal LTP, likely due to the KOR-mediated reduction in DA release from the nigrostriatal pathway (57) (Table 1).

## Opioid neurontransmission in parkinson's disease and levodopa-induced dyskinesia

The broad distribution of opioid receptors in the striatum and their interplay with dopaminergic transmission point at critical role for opioidergic neuropeptides in modulating striatal activity and motor control, in particular, both in healthy and pathological states, such as in PD. This is a progressive neurodegenerative disorder characterized by the loss of dopaminergic cells in the SNpc, which results in motor deficits (i.e., bradykinesia, rest tremor, rigidity, and postural and gait impairment) (65, 66). PD patients develop these symptoms only after a significant depletion of striatal DA– by 60 to 80% (67) – likely because of compensatory DA production by surviving neurons or unknown compensatory mechanisms within or outside of the BG (68). Furthermore, the loss of dopaminergic neurons in the SNpc results in a functional imbalance in the two major output pathways of the striatum: hypoactivity in the direct circuit and hyperactivity in the indirect circuit. This imbalance leads to an overstimulation of the GPi/SNpr which decreases thalamic input to motor cortical areas, resulting in reduced movement and classical Parkinson's symptoms (Figure 1B).

Various animal models of PD are used to better understand the disease's pathophysiology, but none of them fully exhibit all PD symptoms, nor do they develop a neurodegenerative state similar to that in PD patients. For this reason, the most suitable animal model depends on the scientific question being investigated (69). Parkinsonian motor deficits due to DA depletion or DA neuronal death are usually recreated in animals through the injection of selective neurotoxins such as 6-hydroxydopamine (6-OHDA) in rat and 1-methyl-4-phenyl-1,2,3,6-tetrahydropyridine (MPTP) in mouse or primate, while specific molecular events and protein aggregation are investigated using genetic models of PD-related mutations (70).

PD symptoms can be alleviated with dopaminergic treatments that aim to replace the DA deficiency in the nigrostriatal pathway. No curative treatments exist for PD patients, and currently available therapies are symptomatic. To date, L-DOPA remains the most effective drug for exogenous dopaminergic replacement and for counteracting PD symptoms. However, as the disease progresses and dosages of L-DOPA increase, many patients develop disabling complications, including severe fluctuations in motor function (on-off phenomena) and abnormal involuntary movements called L-DOPA-induced dyskinesia (LID) (71, 72). The pathophysiology of LID has been associated with aberrant activation of the direct striatal pathway and with increased levels of the endogenous opioid neuropeptides Enk and Dyn (Figure 1C). It is still unclear whether opioid transmission can affect the neural reorganization of striatal pathways, and if changes in opioid expression might have a compensatory or synergistic effect on striatal-based behaviors in PD and LID.

## Opioid peptide expression in PD and LID

Several studies have been conducted in animal models to investigate changes in the expression of endogenous opioids in the dorsal striatum and their association with motor impairment and dyskinetic movements. Indeed, DA and its binding to D1Rs and D2Rs can modulate the striatal levels of mRNA expression of Dyn and Enk neuropeptide precursors (PDYN and PPENK). Changes in PDYN and PPENK mRNA levels have been observed in PD, where DA transmission is lost, and in LID, during the exogenous replacement of DA (Figure 1).

In the striatum of 6-OHDA and MPTP animal models (60, 62, 73, 74), as well as in PD patients (75), the levels of PPENK mRNA expression are increased, irrespective of L-DOPA treatment. The levels of PPENK mRNA remain highly expressed in PD animals also given chronic L-DOPA treatment (76) as well as in PD patients affected by dyskinesia (77), suggesting persistent adaptive changes in the Enk peptide (78).

In contrast, nigrostriatal DA denervation leads to a reduction in the levels of PDYN mRNA (60, 62, 79, 80) that increase under L-DOPA therapy compared to untreated or non-dyskinetic states, consistently across different study models (62, 76, 78, 81). These observations suggest that the expression of opioidergic neuropeptides involved in the modulation of BG output is strictly regulated by striatal DA levels, likely also through the activation of postsynaptic transcription factors that ultimately can promote the expression of multiple genes, including those for opioidergic peptides.

In addition, in dyskinetic PD rat model, high levels of both PPENK and PDYN are overall correlated with L-DOPA-induced locomotor alterations. While there is a more specific association between high levels of PDYN mRNA and dyskinetic movements (60), on the other hand, high expression of PPENK mRNA is also correlated with locomotor hyperactivity, beyond dyskinesia (62). These observations suggest that Enk and Dyn might play different roles in striatal-based behavioral effects and in locomotor alterations in response to dopaminergic treatment.

## Opioid receptor expression in PD and LID

Along with different levels of opioidergic peptides expression, alterations in the levels of opioidergic receptor immunoreactivity have been observed in both PD patients and animal models. Piccini et al. (82) found reduced opioid receptor binding in the caudate of PD patients, and in the putamen and thalamus of dyskinetic PD patients compared to non-dyskinetic. Similar observations have been described in animal studies, although some differences were found across the various models.

Striatal levels of MOR binding and μ-immunoreactivity were reduced in PD rats (83) and in MPTP-lesioned macaques treated with L-DOPA (80), as well as in PD patients undergoing chronic L-DOPA therapy (84). Lower levels of DOR binding are expressed in the GP and striatal areas of 6-OHDA dyskinetic rats, while an increase of δ-immunoreactivity occurs in the motor and premotor cortex (83) (Figure 2). Consistent with these results, PD patients treated with L-DOPA have reduced levels of DOR binding compared with control patients (84). KOR binding levels are decreased in the striatal areas of dyskinetic PD rats and in the GP of PD rats with and without LID (83); low κ-immunoreactivity is observed only in the GP structure of MPTP-denervated macaques with and without dyskinesia (80).

Even though the exposure to L-DOPA treatment in PD animals and PD patients leads to a reduction in opioid receptor binding levels, Chen and colleagues (85) assessed G protein-coupled receptor signaling as a marker of MOR, DOR and KOR activity in MPTP-lesioned non-human primates. Interestingly, they found a hyperactive transduction signal mediated by all three opioid receptors in the striatum. This suggests that in the parkinsonian state under L-DOPA treatment, although the levels of receptor binding can be decreased, the response to activation of opioid receptors is in fact enhanced.

## Pharmacological implications of opioids in motor function

Elucidating the role of opioidergic transmission in the molecular mechanisms that control motor function is complex, not only due to the striatum's neural heterogeneity, but also because of the broad distribution of opioid receptors throughout the brain. The activation of opioid-mediated postsynaptic signaling cascades likely depends on several factors, including opioid agonists and their response to ORs, the type of ORs activated, and whether receptor stimulation is acute or chronic. Systemic administration of opioidergic drugs might affect different neural circuits and modulate behavioral aspects beyond locomotor activity. Therefore, pharmacological approaches used to distinguish the neural pathways in the control and alteration of movement should be considered critically.

Considering the enhanced expression of endogenous opioid peptides in the striatum of PD animal models and in PD patients, selective agonists and antagonists to ORs have been used to counteract akinesia in PD and to reduce the development of dyskinesia in response to L-DOPA treatment (Table 2). MOR antagonists (cyprodine and ADL5510) alleviated LID in MPTP-lesioned non-human primates without interfering with the antiparkinsonian effects of L-DOPA (86, 87). A selective DOR antagonist (naltrindole) has a similar effect, reducing dyskinetic movements in MPTP-lesioned marmoset and 6-OHDA rats treated with L-DOPA (86, 88), although there is an akinetic effect on motor activity in a PD model without DA treatment (89). A selective DOR agonist (SNC-80) increased locomotor activity in naive and PD animals, but its potential therapeutic applications are limited by its convulsive effects (90–92). In contrast, a κ-receptor antagonist (norBNI) did not induce any anti-dyskinetic effect in MPTP-lesioned macaques (86); yet a selective κ-receptor agonist (U50, 488) reduced LID in PD rats and monkeys, but impaired the anti-parkinsonian effects of L-DOPA treatment (93). In line with these effects, the synthetic opioid analgesic nalbuphine, acting as both a KOR agonist and a MOR antagonist, reduced LID in a non-human primate model of PD and decreased the levels of specific molecular markers associated with the development of dyskinesia (94). Also noteworthy is the effect of the non-selective antagonist naloxone, which reduced LID in 6-OHDA rats (95, 96), although results in MPTP-lesioned macaques and PD patients were inconclusive (97, 98).

**Table 2:** Summary of opioidergic drugs used as pharmacological intervention to counteract parkinsonian symptoms and dyskinetic movements in PD animal model.

**Opioidergic drugs**	**Opioid receptor targets**	**Function**	**Effect**
Cyprodine ADL5510	MOR	Antagonist	↓ LID (86, 87)
Naltrindole	DOR	Antagonist	↓ LID (86, 88) Akinesia (89)
SNC-80	DOR	Agonist	↑ Kinesia in PD state (90–92)
nor-BNI	KOR	Antagonist	No effect on LID (86)
U50,488	KOR	Agonist	↓ LID (93) ↑ Akinesia
Nalbuphine	KOR-MOR	Agonist-antagonist	↓ LID (94)
Naloxone	KOR-MOR-DOR	Antagonist	↓ LID (95, 96)

*nor-BNI, nor-binaltorphimine; LID, levodopa-induced dyskinesia; PD, Parkinson disease*.

The literature makes it clear that different pharmacological responses are expected across animal models and in human patients, likely due to the greater neural organization and connectivity in primates and humans. The lost of DA in PD and its exogenous replacement by L-DOPA lead to changes in the expression of opioid peptides and receptor immunoreactivity that reflect a strong interaction between dopaminergic and opioidergic systems in the BG motor circuit. However, it is still debated whether changes in the opioid transmission occur to compensate for DA denervation and L-DOPA treatment, or whether these changes interact with the molecular and synaptic mechanisms associated with altered neural responses in motor diseases.

## Concluding remarks

The recent advances in understanding the striatal functionality highlight the strong impact of opioidergic transmission to modulate synaptic plasticity and cellular responses of the SPNs. The studies here reviewed, demonstrate that opioid receptors have a regional (ventral vs. dorsal striatum), compartmental (striosomes vs. matrix) and cellular (dSPNs vs. iSPNs) specificity that affects the striatal activity in response to different inputs. Such specificity reflects the complexity of striatal organization and the efforts to find selective opioidergic treatments that can modulate specific neural pathways. Although the literature points out the inhibitory effect of opioid agonists on synaptic transmission and neurotransmitters release, it is still debated how opioid receptors interact with dopaminergic receptors and whether they share common mechanisms to activate postsynaptic signaling cascades and downstream effectors. The interaction between opioidergic and dopaminergic pathways becomes crucial in PD and LID where the high levels of endogenous opioids occurs in parallel with aberrant dopaminergic transmission, and are associated with alterated striatal-based behaviors. Since the broad distribution of opioid receptors throughout the brain, pharmacological approaches should aim to selectively target defined receptor subtypes, in a cell-type- and input-specific manner. The use of chemogenetic or optogenetic approaches are therefore crucial to dissect opioidergic neurotransmission within the striatum and its interaction with dopaminergic system. This would be instrumental to develop specific pharmacological treatments able to restore maladaptive changes without interfering with other neuronal pathways.

## Author contributions

SS wrote the manuscript. RT conceived the review contribution, supervised the writing and critically edited the manuscript.

### Conflict of interest statement

The authors declare that the research was conducted in the absence of any commercial or financial relationships that could be construed as a potential conflict of interest. The reviewer GF declared a past collaboration with one of the authors RT to the handling Editor.

## References

[1] Le Merrer J Becker JA Befort K Kieffer BL. Reward processing by the opioid system in the brain. Physiol Rev (2009) 89:1379–412. 10.1152/physrev.00005.2009 19789384 PMC4482114

[2] Lutz PE Kieffer BL. Opioid receptors: distinct roles in mood disorders. Trends Neurosci. (2013) 36:195–206. 10.1016/j.tins.2012.11.002 23219016 PMC3594542

[3] Crossman AR. Neural mechanisms in disorders of movement. Comp Biochem Physiol Comp Physiol. (1989) 93:141–9. 2568216 10.1016/0300-9629(89)90201-6

[4] DeLong MR. Primate models of movement disorders of basal ganglia origin. Trends Neurosci (1990) 13:281–5. 1695404 10.1016/0166-2236(90)90110-v

[5] Redgrave P Rodriguez M Smith Y Rodriguez-Oroz MC Lehericy S Bergman H. Goal-directed and habitual control in the basal ganglia: implications for Parkinson's disease. Nat Rev Neurosci. (2010) 11:760–72. 10.1038/nrn2915 20944662 PMC3124757

[6] Lei W Jiao Y Del Mar N Reiner A. Evidence for differential cortical input to direct pathway versus indirect pathway striatal projection neurons in rats. J Neurosci. (2004) 24:8289–99. 10.1523/JNEUROSCI.1990-04.2004 15385612 PMC6729697

[7] Barroso-Chinea P Bezard E. Basal Ganglia circuits underlying the pathophysiology of levodopa-induced dyskinesia. Front Neuroanat. (2010) 4:131. 10.3389/fnana.2010.00131 20890450 PMC2947938

[8] Obeso JA Rodriguez-Oroz MC Benitez-Temino B Blesa FJ Guridi J Marin C. Functional organization of the basal ganglia: therapeutic implications for Parkinson's disease. Mov Disord. (2008) 23 Suppl. 3:S548–59. 10.1002/mds.22062 18781672

[9] Gerfen CR Young WS III. Distribution of striatonigral and striatopallidal peptidergic neurons in both patch and matrix compartments: an in situ hybridization histochemistry and fluorescent retrograde tracing study. Brain Res (1988) 460:161–7. 2464402 10.1016/0006-8993(88)91217-6

[10] Fallon JH Leslie FM Cone RI. Dynorphin-containing pathways in the substantia nigra and ventral tegmentum: a double labeling study using combined immunofluorescence and retrograde tracing. Neuropeptides (1985) 5:457–60. 2860605 10.1016/0143-4179(85)90053-8

[11] Cuello AC Paxinos G. Evidence for a long Leu-enkephalin striopallidal pathway in rat brain. Nature (1978) 271:178–80. 23498 10.1038/271178a0

[12] Albin RL Young AB Penney JB. The functional anatomy of basal ganglia disorders. Trends Neurosci. (1989) 12:366–75. 2479133 10.1016/0166-2236(89)90074-x

[13] Obeso JA Marin C Rodriguez-Oroz C Blesa J Benitez-Temino B Mena-Segovia J. The basal ganglia in Parkinson's disease: current concepts and unexplained observations. Ann Neurol. (2008) 64 Suppl. 2:S30–46. 10.1002/ana.21481 19127584

[14] Cui G Jun SB Jin X Pham MD Vogel SS Lovinger DM. Concurrent activation of striatal direct and indirect pathways during action initiation. Nature (2013) 494:238–42. 10.1038/nature11846 23354054 PMC4039389

[15] Rice ME Cragg SJ. Dopamine spillover after quantal release: rethinking dopamine transmission in the nigrostriatal pathway. Brain Res Rev. (2008) 58:303–13. 10.1016/j.brainresrev.2008.02.004 18433875 PMC2879278

[16] Liu C Kershberg L Wang J Schneeberger S Kaeser PS. Dopamine secretion is mediated by sparse active zone-like release sites. Cell (2018) 172:706–18e15. 10.1016/j.cell.2018.01.008 29398114 PMC5807134

[17] West AR Grace AA. Opposite influences of endogenous dopamine D1 and D2 receptor activation on activity states and electrophysiological properties of striatal neurons: studies combining *in vivo* intracellular recordings and reverse microdialysis. J. Neurosci. (2002) 22:294–304. 10.1523/JNEUROSCI.22-01-00294.2002 11756513 PMC6757590

[18] da Silva JA Tecuapetla F Paixao V Costa RM Dopamine neuron activity before action initiation gates and invigorates future movements. Nature (2018) 554:244–8. 10.1038/nature25457 29420469

[19] Williams JT Christie MJ Manzoni O. Cellular and synaptic adaptations mediating opioid dependence. Physiol Rev. (2001) 81:299–343. 10.1152/physrev.2001.81.1.299 11152760

[20] Dacher M Nugent FS. Opiates and plasticity. Neuropharmacology (2011) 61:1088–96. 10.1016/j.neuropharm.2011.01.028 21272593

[21] Smith AP Lee NM. Opioid receptor interactions: local and nonlocal, symmetric and asymmetric, physical and functional. Life Sci. (2003) 73:1873–93. 10.1016/S0024-3205(03)00549-6 12899914

[22] Atwood BK Kupferschmidt DA Lovinger DM. Opioids induce dissociable forms of long-term depression of excitatory inputs to the dorsal striatum. Nat Neurosci. (2014) 17:540–8. 10.1038/nn.3652 24561996 PMC4163916

[23] Tajima K Fukuda T. Region-specific diversity of striosomes in the mouse striatum revealed by the differential immunoreactivities for mu-opioid receptor, substance P, and enkephalin. Neuroscience (2013) 241:215–28. 10.1016/j.neuroscience.2013.03.012 23518224

[24] Oude Ophuis RJ Boender AJ van Rozen AJ Adan RA Cannabinoid, melanocortin and opioid receptor expression on DRD1 and DRD2 subpopulations in rat striatum. Front Neuroanat (2014) 8:14 10.3389/fnana.2014.00014 24723856 PMC3972466

[25] Ambrose LM Unterwald EM Van Bockstaele EJ. Ultrastructural evidence for co-localization of dopamine D2 and micro-opioid receptors in the rat dorsolateral striatum. Anat Record Part A Discov Mol Cell Evol Biol (2004) 279:583–91. 10.1002/ar.a.20054 15224400

[26] Ponterio G Tassone A Sciamanna G Riahi E Vanni V Bonsi P. Powerful inhibitory action of mu opioid receptors (MOR) on cholinergic interneuron excitability in the dorsal striatum. Neuropharmacology (2013) 75:78–85. 10.1016/j.neuropharm.2013.07.006 23891638

[27] Jabourian M Venance L Bourgoin S Ozon S Perez S Godeheu G. Functional mu opioid receptors are expressed in cholinergic interneurons of the rat dorsal striatum: territorial specificity and diurnal variation. Eur J Neurosci. (2005) 21:3301–9. 10.1111/j.1460-9568.2005.04154.x 16026468

[28] Cahill CM McClellan KA Morinville A Hoffert C Hubatsch D O'Donnell D. Immunohistochemical distribution of delta opioid receptors in the rat central nervous system: evidence for somatodendritic labeling and antigen-specific cellular compartmentalization. J Comp Neurol (2001) 440:65–84. 10.1002/cne.1370 11745608

[29] Banghart MR Neufeld SQ Wong NC Sabatini BL. Enkephalin disinhibits mu opioid receptor-rich striatal patches via delta opioid receptors. Neuron (2015) 88:1227–39. 10.1016/j.neuron.2015.11.010 26671460 PMC4698909

[30] North RA Williams JT Surprenant A Christie MJ. Mu and delta receptors belong to a family of receptors that are coupled to potassium channels. Proc Natl Acad Sci USA. (1987) 84:5487–91. 2440052 10.1073/pnas.84.15.5487 PMC298884

[31] Al-Hasani R Bruchas MR. Molecular mechanisms of opioid receptor-dependent signaling and behavior. Anesthesiology (2011) 115:1363–81. 10.1097/ALN.0b013e318238bba6 22020140 PMC3698859

[32] Mansour A Fox CA Akil H Watson SJ. Opioid-receptor mRNA expression in the rat CNS: anatomical and functional implications. Trends Neurosci. (1995) 18:22–9. 7535487 10.1016/0166-2236(95)93946-u

[33] Kivell B Uzelac Z Sundaramurthy S Rajamanickam J Ewald A Chefer V. Salvinorin A regulates dopamine transporter function via a kappa opioid receptor and ERK1/2-dependent mechanism. Neuropharmacology (2014) 86:228–40. 10.1016/j.neuropharm.2014.07.016 25107591 PMC4188751

[34] Hipolito L Sanchez-Catalan MJ Zanolini I Polache A Granero L. Shell/core differences in mu- and delta-opioid receptor modulation of dopamine efflux in nucleus accumbens. Neuropharmacology (2008) 55:183–9. 10.1016/j.neuropharm.2008.05.012 18582908

[35] Campos-Jurado Y Marti-Prats L Zornoza T Polache A Granero L Cano-Cebrian MJ. Regional differences in mu-opioid receptor-dependent modulation of basal dopamine transmission in rat striatum. Neurosci Lett. (2017) 638:102–8. 10.1016/j.neulet.2016.12.024 27986497

[36] Costa-Neto CM Parreiras ESLT Bouvier M. A Pluridimensional View of Biased Agonism. Mol Pharmacol. (2016) 90:587–95. 10.1124/mol.116.105940 27638872

[37] Reiter E Ahn S Shukla AK Lefkowitz RJ. Molecular mechanism of beta-arrestin-biased agonism at seven-transmembrane receptors. Annu Rev Pharmacol Toxicol. (2012) 52:179–97. 10.1146/annurev.pharmtox.010909.105800 21942629 PMC3628752

[38] Pradhan AA Becker JA Scherrer G Tryoen-Toth P Filliol D Matifas A. *In vivo* delta opioid receptor internalization controls behavioral effects of agonists. PLoS ONE (2009) 4:e5425. 10.1371/journal.pone.0005425 19412545 PMC2672171

[39] Kramer HK Onoprishvili I Andria ML Hanna K Sheinkman K Haddad LB Delta opioid activation of the mitogen-activated protein kinase cascade does not require transphosphorylation of receptor tyrosine kinases. BMC Pharmacol. (2002) 2:5 10.1186/1471-2210-2-5 11897012 PMC88976

[40] Valjent E Pascoli V Svenningsson P Paul S Enslen H Corvol JC. Regulation of a protein phosphatase cascade allows convergent dopamine and glutamate signals to activate ERK in the striatum. Proc Natl Acad Sci USA. (2005) 102:491–6. 10.1073/pnas.0408305102 15608059 PMC544317

[41] Belcheva MM Clark AL Haas PD Serna JS Hahn JW Kiss A. Mu and kappa opioid receptors activate ERK/MAPK via different protein kinase C isoforms and secondary messengers in astrocytes. J Biol Chem. (2005) 280:27662–9. 10.1074/jbc.M502593200 15944153 PMC1400585

[42] Cenci MA Lundblad M. Post- versus presynaptic plasticity in L-DOPA-induced dyskinesia. J Neurochem. (2006) 99:381–92. 10.1111/j.1471-4159.2006.04124.x 16942598

[43] Westin JE Vercammen L Strome EM Konradi C Cenci MA. Spatiotemporal pattern of striatal ERK1/2 phosphorylation in a rat model of L-DOPA-induced dyskinesia and the role of dopamine D1 receptors. Biol Psychiatry (2007) 62:800–10. 10.1016/j.biopsych.2006.11.032 17662258 PMC4205578

[44] Santini E Valjent E Usiello A Carta M Borgkvist A Girault JA. Critical involvement of cAMP/DARPP-32 and extracellular signal-regulated protein kinase signaling in L-DOPA-induced dyskinesia. J Neurosci. (2007) 27:6995–7005. 10.1523/JNEUROSCI.0852-07.2007 17596448 PMC6672217

[45] Fasano S Bezard E D'Antoni A Francardo V Indrigo M Qin L. Inhibition of Ras-guanine nucleotide-releasing factor 1 (Ras-GRF1) signaling in the striatum reverts motor symptoms associated with L-dopa-induced dyskinesia. Proc Natl Acad Sci USA. (2010) 107:21824–9. 10.1073/pnas.1012071107 21115823 PMC3003069

[46] Lopez-Huerta VG Nakano Y Bausenwein J Jaidar O Lazarus M Cherassse Y. Erratum to: The neostriatum: two entities, one structure? Brain Struct Funct. (2016) 221:1789. 10.1007/s00429-015-1017-8 25754484 PMC4969893

[47] Graybiel AM. Neurotransmitters and neuromodulators in the basal ganglia. Trends Neurosci. (1990) 13:244–54. 1695398 10.1016/0166-2236(90)90104-i

[48] Miura M Saino-Saito S Masuda M Kobayashi K Aosaki T. Compartment-specific modulation of GABAergic synaptic transmission by mu-opioid receptor in the mouse striatum with green fluorescent protein-expressing dopamine islands. J Neurosci. (2007) 27:9721–8. 10.1523/JNEUROSCI.2993-07.2007 17804632 PMC6672981

[49] Graybiel AM Ragsdale CW Jr. Histochemically distinct compartments in the striatum of human, monkeys, and cat demonstrated by acetylthiocholinesterase staining. Proc Natl Acad Sci USA. (1978) 75:5723–6. 103101 10.1073/pnas.75.11.5723 PMC393041

[50] Gerfen CR. The neostriatal mosaic: compartmentalization of corticostriatal input and striatonigral output systems. Nature (1984) 311:461–4. 6207434 10.1038/311461a0

[51] Gerfen CR Baimbridge KG Miller JJ. The neostriatal mosaic: compartmental distribution of calcium-binding protein and parvalbumin in the basal ganglia of the rat and monkey. Proc Natl Acad Sci USA. (1985) 82:8780–4. 3909155 10.1073/pnas.82.24.8780 PMC391521

[52] Fujiyama F Sohn J Nakano T Furuta T Nakamura KC Matsuda W. Exclusive and common targets of neostriatofugal projections of rat striosome neurons: a single neuron-tracing study using a viral vector. Eur J Neurosci. (2011) 33:668–77. 10.1111/j.1460-9568.2010.07564.x 21314848

[53] Crittenden JR Graybiel AM. Basal Ganglia disorders associated with imbalances in the striatal striosome and matrix compartments. Front Neuroanat. (2011) 5:59. 10.3389/fnana.2011.00059 21941467 PMC3171104

[54] Brimblecombe KR Cragg SJ. The striosome and matrix compartments of the striatum: a path through the labyrinth from neurochemistry toward function. ACS Chem Neurosci. (2017) 8:235–42. 10.1021/acschemneuro.6b00333 27977131

[55] Jiang ZG North RA. Pre- and postsynaptic inhibition by opioids in rat striatum. J Neurosci. (1992) 12:356–61. 1309576 10.1523/JNEUROSCI.12-01-00356.1992 PMC6575700

[56] Blomeley CP Bracci E. Opioidergic interactions between striatal projection neurons. J Neurosci. (2011) 31:13346–56. 10.1523/JNEUROSCI.1775-11.2011 21940429 PMC3781771

[57] Hawes SL Salinas AG Lovinger DM Blackwell KT. Long-term plasticity of corticostriatal synapses is modulated by pathway-specific co-release of opioids through kappa-opioid receptors. J Physiol. (2017) 595:5637–52. 10.1113/JP274190 28449351 PMC5556153

[58] Pisa M. Motor functions of the striatum in the rat: critical role of the lateral region in tongue and forelimb reaching. Neuroscience (1988) 24:453–63. 3362348 10.1016/0306-4522(88)90341-7

[59] Andersson M Hilbertson A Cenci MA. Striatal fosB expression is causally linked with l-DOPA-induced abnormal involuntary movements and the associated upregulation of striatal prodynorphin mRNA in a rat model of Parkinson's disease. Neurobiol Dis. (1999) 6:461–74. 10.1006/nbdi.1999.0259 10600402

[60] Cenci MA Lee CS Bjorklund A. L-DOPA-induced dyskinesia in the rat is associated with striatal overexpression of prodynorphin- and glutamic acid decarboxylase mRNA. Eur J Neurosci. (1998) 10:2694–706. 9767399

[61] Capper-Loup C Frey CM Rebell D Kaelin-Lang A. Adaptive gene expression changes on the healthy side of parkinsonian rats. Neuroscience (2013) 233:157–65. 10.1016/j.neuroscience.2012.12.027 23270858

[62] Sgroi S Capper-Loup C Paganetti P Kaelin-Lang A. Enkephalin and dynorphin neuropeptides are differently correlated with locomotor hypersensitivity and levodopa-induced dyskinesia in parkinsonian rats. Exp Neurol. (2016) 280:80–8. 10.1016/j.expneurol.2016.03.024 27072528

[63] Nugent FS Penick EC Kauer JA. Opioids block long-term potentiation of inhibitory synapses. Nature (2007) 446:1086–90. 10.1038/nature05726 17460674

[64] Drdla R Gassner M Gingl E Sandkuhler J. Induction of synaptic long-term potentiation after opioid withdrawal. Science (2009) 325:207–10. 10.1126/science.1171759 19590003

[65] Massano J Bhatia KP. Clinical approach to Parkinson's disease: features, diagnosis, and principles of management. Cold Spring Harbor Perspect Med (2012) 2:a008870. 10.1101/cshperspect.a008870 22675666 PMC3367535

[66] Sgroi S Kaelin-Lang A Capper-Loup C Spontaneous locomotor activity and L-DOPA-induced dyskinesia are not linked in 6-OHDA parkinsonian rats. Front Behav Neurosci. (2014) 8:331 10.3389/fnbeh.2014.00331 25324746 PMC4183109

[67] Bernheimer H Birkmayer W Hornykiewicz O Jellinger K Seitelberger F. Brain dopamine and the syndromes of Parkinson and Huntington. Clinical, morphological and neurochemical correlations. J Neurol Sci. (1973) 20:415–55. 4272516 10.1016/0022-510x(73)90175-5

[68] Bezard E Gross CE Brotchie JM Presymptomatic compensation in Parkinson's disease is not dopamine-mediated. Trends Neurosci (2003) 26:215–21. 10.1016/S0166-2236(03)00038-9 12689773

[69] Cenci MA Whishaw IQ Schallert T. Animal models of neurological deficits: how relevant is the rat? Nat Rev Neurosci. (2002) 3:574–9. 10.1038/nrn877 12094213

[70] Blesa J Phani S Jackson-Lewis V Przedborski S. Classic and new animal models of Parkinson's disease. J Biomed Biotechnol. (2012) 2012:845618. 10.1155/2012/845618 22536024 PMC3321500

[71] Granerus AK. Factors influencing the occurrence of “on-off” symptoms during long-term treatment with L-dopa. Acta Med Scand. (1978) 203:75–85. 626117 10.1111/j.0954-6820.1978.tb14835.x

[72] Fabbrini G Brotchie JM Grandas F Nomoto M Goetz CG. Levodopa-induced dyskinesias. Mov Disord. (2007) 22:1379–89. 10.1002/mds.21475 17427940

[73] Gerfen CR Engber TM Mahan LC Susel Z Chase TN Monsma FJ Jr. D1 and D2 dopamine receptor-regulated gene expression of striatonigral and striatopallidal neurons. Science (1990) 250:1429–32. 2147780 10.1126/science.2147780

[74] Herrero MT Augood SJ Hirsch EC Javoy-Agid F Luquin MR Agid Y. Effects of L-DOPA on preproenkephalin and preprotachykinin gene expression in the MPTP-treated monkey striatum. Neuroscience (1995) 68:1189–98. 8544992 10.1016/0306-4522(95)00120-8

[75] Nisbet AP Foster OJ Kingsbury A Eve DJ Daniel SE Marsden CD. Preproenkephalin and preprotachykinin messenger RNA expression in normal human basal ganglia and in Parkinson's disease. Neuroscience (1995) 66:361–76. 7477878 10.1016/0306-4522(94)00606-6

[76] Westin JE Andersson M Lundblad M Cenci MA. Persistent changes in striatal gene expression induced by long-term L-DOPA treatment in a rat model of Parkinson's disease. Eur J Neurosci (2001) 14:1171–6. 10.1046/j.0953-816x.2001.01743.x 11683909

[77] Calon F Birdi S Rajput AH Hornykiewicz O Bedard PJ Di Paolo T. Increase of preproenkephalin mRNA levels in the putamen of Parkinson disease patients with levodopa-induced dyskinesias. J Neuropathol Exp Neurol. (2002) 61:186–96. 10.1093/jnen/61.2.186 11853020

[78] Tamim MK Samadi P Morissette M Gregoire L Ouattara B Levesque D. Effect of non-dopaminergic drug treatment on Levodopa induced dyskinesias in MPTP monkeys: common implication of striatal neuropeptides. Neuropharmacology (2010) 58:286–96. 10.1016/j.neuropharm.2009.06.030 19576910

[79] Tel BC Zeng BY Cannizzaro C Pearce RK Rose S Jenner P. Alterations in striatal neuropeptide mRNA produced by repeated administration of L-DOPA, ropinirole or bromocriptine correlate with dyskinesia induction in MPTP-treated common marmosets. Neuroscience (2002) 115:1047–58. 10.1016/S0306-4522(02)00535-3 12453478

[80] Aubert I Guigoni C Li Q Dovero S Bioulac BH Gross CE. Enhanced preproenkephalin-B-derived opioid transmission in striatum and subthalamic nucleus converges upon globus pallidus internalis in L-3,4-dihydroxyphenylalanine-induced dyskinesia. Biol Psychiatry (2007) 61:836–44. 10.1016/j.biopsych.2006.06.038 16950226

[81] Bishop C Krolewski DM Eskow KL Barnum CJ Dupre KB Deak T. Contribution of the striatum to the effects of 5-HT1A receptor stimulation in L-DOPA-treated hemiparkinsonian rats. J Neurosci Res. (2009) 87:1645–58. 10.1002/jnr.21978 19115412 PMC2670562

[82] Piccini P Weeks RA Brooks DJ. Alterations in opioid receptor binding in Parkinson's disease patients with levodopa-induced dyskinesias. Ann Neurol. (1997) 42:720–6. 10.1002/ana.410420508 9392571

[83] Johansson PA Andersson M Andersson KE Cenci MA. Alterations in cortical and basal ganglia levels of opioid receptor binding in a rat model of l-DOPA-induced dyskinesia. Neurobiol Dis. (2001) 8:220–39. 10.1006/nbdi.2000.0372 11300719

[84] Fernandez A de Ceballos ML Jenner P Marsden CD. Neurotensin, substance P, delta and mu opioid receptors are decreased in basal ganglia of Parkinson's disease patients. Neuroscience (1994) 61:73–9. 7969897 10.1016/0306-4522(94)90061-2

[85] Chen L Togasaki DM Langston JW Di Monte DA Quik M. Enhanced striatal opioid receptor-mediated G-protein activation in L-DOPA-treated dyskinetic monkeys. Neuroscience (2005) 132:409–20. 10.1016/j.neuroscience.2004.10.026 15802193

[86] Henry B Fox SH Crossman AR Brotchie JM. Mu- and delta-opioid receptor antagonists reduce levodopa-induced dyskinesia in the MPTP-lesioned primate model of Parkinson's disease. Exp Neurol. (2001) 171:139–46. 10.1006/exnr.2001.7727 11520128

[87] Koprich JB Fox SH Johnston TH Goodman A Le Bourdonnec B Dolle RE. The selective mu-opioid receptor antagonist ADL5510 reduces levodopa-induced dyskinesia without affecting antiparkinsonian action in MPTP-lesioned macaque model of Parkinson's disease. Mov Disord. (2011) 26:1225–33. 10.1002/mds.23631 21465551

[88] Billet F Costentin J Dourmap N. Influence of corticostriatal delta-opioid receptors on abnormal involuntary movements induced by L-DOPA in hemiparkinsonian rats. Exp Neurol. (2012) 236:339–50. 10.1016/j.expneurol.2012.04.017 22575599

[89] Mabrouk OS Volta M Marti M Morari M. Stimulation of delta opioid receptors located in substantia nigra reticulata but not globus pallidus or striatum restores motor activity in 6-hydroxydopamine lesioned rats: new insights into the role of delta receptors in parkinsonism. J Neurochem. (2008) 107:1647–59. 10.1111/j.1471-4159.2008.05727.x 19094058

[90] Spina L Longoni R Mulas A Chang KJ Di Chiara G. Dopamine-dependent behavioural stimulation by non-peptide delta opioids BW373U86 and SNC 80: 1. Locomotion, rearing and stereotypies in intact rats. Behav Pharmacol. (1998) 9:1–8. 9832942

[91] Hudzik TJ Howell A Payza K Cross AJ. Antiparkinson potential of delta-opioid receptor agonists. Eur J Pharmacol. (2000) 396:101–7. 10.1016/S0014-2999(00)00209-0 10822062

[92] Nozaki C Le Bourdonnec B Reiss D Windh RT Little PJ Dolle RE. delta-Opioid mechanisms for ADL5747 and ADL5859 effects in mice: analgesia, locomotion, and receptor internalization. J Pharmacol Exp Ther. (2012) 342:799–807. 10.1124/jpet.111.188987 22700431 PMC3422521

[93] Cox H Togasaki DM Chen L Langston JW Di Monte DA Quik M. The selective kappa-opioid receptor agonist U50,488 reduces L-dopa-induced dyskinesias but worsens parkinsonism in MPTP-treated primates. Exp Neurol. (2007) 205:101–7. 10.1016/j.expneurol.2007.01.024 17335811 PMC2001245

[94] Potts LF Park ES Woo JM Dyavar Shetty BL Singh A Braithwaite SP. Dual kappa-agonist/mu-antagonist opioid receptor modulation reduces levodopa-induced dyskinesia and corrects dysregulated striatal changes in the nonhuman primate model of Parkinson disease. Ann Neurol. (2015) 77:930–41. 10.1002/ana.24375 25820831 PMC6235675

[95] Lundblad M Andersson M Winkler C Kirik D Wierup N Cenci MA. Pharmacological validation of behavioural measures of akinesia and dyskinesia in a rat model of Parkinson's disease. Eur J Neurosci. (2002) 15:120–32. 10.1046/j.0953-816x.2001.01843.x 11860512

[96] Klintenberg R Svenningsson P Gunne L Andren PE. Naloxone reduces levodopa-induced dyskinesias and apomorphine-induced rotations in primate models of parkinsonism. J Neural Trans. (2002) 109:1295–307. 10.1007/s00702-002-0715-6 12373562

[97] Gomez-Mancilla B Bedard PJ. Effect of nondopaminergic drugs on L-dopa-induced dyskinesias in MPTP-treated monkeys. Clin Neuropharmacol. (1993) 16:418–27. 8106150 10.1097/00002826-199310000-00004

[98] Fox S Silverdale M Kellett M Davies R Steiger M Fletcher N. Non-subtype-selective opioid receptor antagonism in treatment of levodopa-induced motor complications in Parkinson's disease. Mov Disord. (2004) 19:554–60. 10.1002/mds.10693 15133820

